# Effect of family planning interventions on couple years of protection in Malawi

**DOI:** 10.1002/ijgo.12439

**Published:** 2018-02-02

**Authors:** Clara Lemani, Nenani Kamtuwanje, Billy Phiri, Ilene S. Speizer, Kavita Singh, Olive Mtema, Ndidza Chisanu, Jennifer H. Tang

**Affiliations:** ^1^ University of North Carolina Project‐Malawi Lilongwe Malawi; ^2^ Department of Maternal and Child Health University of North Carolina at Chapel Hill Chapel Hill NC USA; ^3^ Health Policy Project Lilongwe Malawi; ^4^ Family Planning Association of Malawi Lilongwe Malawi; ^5^ Department of Obstetrics and Gynecology University of North Carolina at Chapel Hill Chapel Hill NC USA

**Keywords:** Africa, Couple years of protection, Family planning, Long‐acting reversible contraception, Malawi

## Abstract

**Objective:**

The primary objective was to assess the effect of family planning interventions at two health facilities in Malawi on couple years of protection (CYP).

**Methods:**

A prospective quasi‐experimental design was used to compare CYP and uptake of long‐acting reversible contraception (LARC) between two intervention facilities (Area 25 Health Center and Kasungu District Hospital) and two nonintervention facilities (Mkanda Health Center and Dowa District Hospital). The interventions included community mobilization and demand generation for family planning, and training and mentoring of providers in LARC insertion. Monthly data were collected from 1 year prior to intervention implementation until 2 years thereafter.

**Results:**

From the pre‐intervention year to the second post‐intervention year, CYP increased by 175.1% at Area 25, whereas it decreased by 33.8% at Mkanda. At Kasungu and Dowa, CYP increased by 90.7% and 64.4%, respectively. Uptake of LARC increased by 12.2% at Area 25 r, 6.2% at Kasungu, and 2.9% at Dowa, but decreased by 3.8% at Mkanda.

**Conclusions:**

The interventions led to an increase in CYP and LARC uptake. Future family planning programs should sensitize communities about family planning and train providers to provide all contraceptives so that women can make informed decisions and use the contraceptive of their choice.

## Abstract 2

A package of family planning interventions focused on demand generation and supply‐side training and mentoring increased uptake of contraceptives, particularly long‐acting reversible contraceptives.

## 1 INTRODUCTION

Unintended pregnancy puts women at risk of maternal death and illness. The prevention of unintended pregnancy through increased use of contraceptive methods can help reduce maternal deaths and illness. Malawi, like many countries in Sub‐Saharan Africa, has a low modern‐contraceptive prevalence rate (45%) and a high unmet need for family planning (19%).^1^ The low contraceptive prevalence in Malawi is in part attributable to misconceptions about contraceptive use.^2^, ^3^ Community mobilization can help by correctly educating communities about contraception and could increase the uptake of contraceptive services, especially in rural areas, where the unmet need for family planning is highest.^4^

To accelerate the reduction of maternal and neonatal mortality, Malawi launched the Presidential Initiative on Safe Motherhood in 2012. This initiative has three program interventions: (1) community mobilization and training of local leaders in maternal health and family planning, (2) training of skilled community midwife assistants, and (3) the construction of maternity waiting homes. UNC Project‐Malawi decided to integrate a package of family planning interventions into the three interventions from the Presidential Initiative on Safe Motherhood. The project's primary objective was to increase the couple years of protection (CYP) provided by contraceptive services at two health facilities where UNC Project‐Malawi was constructing maternity waiting homes. “Couple years of protection” is a commonly used family planning metric to monitor output and progress in the delivery of contraceptive services at the project level.^5^, ^6^ Secondary objectives included the introduction of immediate postpartum long‐acting reversible contraception (LARC) and improvement of the facilities' contraceptive method mix.

The present quasi‐experimental study was designed to compare the CYP provided at each of the two intervention health facilities with those provided at two matched nonintervention health facilities for the year before implementation of the family planning package and for the 2 years after the package was implemented.

## 2 MATERIALS AND METHODS

In the present prospective study conducted in Malawi, a package of family planning interventions was implemented at Area 25 Health Center in Lilongwe district and at Kasungu District Hospital. The package included community mobilization and demand generation for family planning, and training and mentoring of health providers in LARC insertion. These activities were implemented specifically for the present study in the two intervention sites only. In addition, two comparison sites were chosen for their similarity to the intervention sites in terms of structure, government oversight, and location in central Malawi. Like the intervention sites, each comparison site also had a recently constructed maternity waiting home. The comparison site for Kasungu District Hospital was Dowa District Hospital, and the comparison site for Area 25 Health Center was Mkanda Health Center in Mchinji district. Data were collected from all four sites for the period between February 1, 2013, and June 30, 2016.

The study was approved by the University of North Carolina (UNC) Institutional Review Board and the Malawi National Health Sciences Research Committee. De‐identified data abstracted from the family planning registers of the four study facilities were analyzed, so no individual consent was required.

UNC Project‐Malawi engaged the Health Policy Project (HPP, funded by the US Agency for International Development) to sensitize religious leaders and their congregations on the benefits of family planning in the catchment areas of the two intervention facilities in order to address barriers to family planning related to religious beliefs. The HPP was already working with the Malawi Ministry of Finance, Economic Planning and Development (MFEPD) to support advocacy for population and family planning issues. They collaborated with the MFEPD to organize a live radio discussion panel (Table 1). The panelists represented the major religions and denominations in Malawi, where approximately 69% of the population are Christian and 26% are Muslim. Only 5% of Malawians subscribe to another or no religion.^7^

**Table 1:** Family planning intervention activities at Area 25 Health Center and Kasungu District Hospital

Activity	Output
Community mobilization/demand creation	
Radio discussion panel with religious leaders	One panel held with national reach
Population weekends with religious leaders	Two population weekends held; one in Area 25, one in Kasungu district
Creation of family planning task forces	Five task forces created; one in Area 25, four in Kasungu district
Establishment of family planning champions	10 champions established; two in Area 25, eight in Kasungu district
Provider training and mentoring	
LARC training courses held	16 training courses held; eight at Area 25 Health Center, eight at Kasungu District Hospital
Providers trained in LARC	101 providers trained; 37 from Area 25 Health Center, 64 in Kasungu District Hospital
Mentors in LARC	14 mentors; seven at Area 25 Health Center, seven at Kasungu District Hospital

Abbreviation: LARC, long‐acting reversible contraception.

The HPP also organized two “population weekends,” one in the catchment area of Area 25 Health Center and another in four areas surrounding Kasungu District Hospital, to promote the use of family planning among the religious faithful. During the population weekends, the participating churches (both Catholic and Protestant) and mosques focused their weekend activities on family planning and rapid population growth and gave out brochures on family planning use and methods; these brochures had been specially designed by each of the three major religious denominations in Malawi (Protestant, Catholic, and Muslim). The HPP also conducted similar activities in other districts of Malawi, but not in the districts of the two comparison sites.

UNC Project‐Malawi also partnered with the Family Planning Association of Malawi (Malawi's International Planned Parenthood Federation affiliate). The association organized open days during the week after each catchment area's population weekend and trained local leaders and community leadership groups in the catchment areas about the benefits of family planning and how to dispel local myths about family planning.

UNC Project‐Malawi sponsored 1‐week training courses on LARC insertion and removal, specifically for implants (Jadelle; Bayer AG, Leverkeusen, Germany, and Implanon Classic; Merck, Kenilworth, NJ, USA) and a copper T380A (Injeflex, Sao Paulo, Brazil) intrauterine contraceptive device (IUD), and 2‐day training courses on immediate postpartum IUD (PPIUD) insertion and removal for providers at the two intervention facilities. These training courses were organized to bridge the gap in LARC provision because it was observed that all providers at the two facilities could provide short‐acting reversible contraception (SARC), but only a few had skills to provide LARC. At the time of the training courses, immediate postpartum implant insertion was not allowed in Malawi outside of research studies. Approval was granted to train providers in both PPIUD and immediate postpartum implant insertion at Kasungu District Hospital through another research study.^8^

Training in LARC continued after the courses through continuous mentoring by providers selected from the two health facilities. Each family planning mentor was assigned to four to six other providers. The family planning mentors were to meet with their mentees on a monthly basis for the 6 months after the training courses and submit their mentees' monthly LARC insertion logs to the project coordinator. In addition, at Area 25 Health Center, 30 community health workers were trained in family planning to scale up the community‐based distribution of SARC and referral to the nearest health facility for LARC insertion and removal.^9^

Monthly data were collected from each facility's family planning register and Malawi's District Health Information Software version 2 (DHIS2) program; family planning data were collected from 1 year prior to the interventions (pre‐intervention data) to 2 years after initiation of the interventions (post‐intervention data). For Area 25 Health Center and Mkanda Health Center, data were collected from February 1, 2013, to January 31, 2016, because the interventions there began in February 2014. For Kasungu District Hospital and Dowa District Hospital, data were collected from July 1, 2013, to June 30, 2016, because the interventions there began in July 2014.

The data collected were the total number of family planning patients; number of new family planning patients; number of new implant or IUD patients; and family planning uptake for each available method, including SARC, LARC, and sterilization. However, data for July 2013 at Area 25 Health Center, January 2016 at Mkanda Health Center, and April 2016 at Kasungu District Hospital were completely missing and were therefore imputed by taking the average for the month before and after the missing month. The data were entered into an Excel (Microsoft, Redmond, WA, USA) spreadsheet.

For the analyses, graphs were created to compare trends in monthly family planning visits and monthly LARC uptake between each intervention site and its comparison site during the 3‐year monitoring period. Then, the absolute difference between each intervention facility and its comparison facility was calculated for each family planning parameter. Finally, CYP were computed for each health facility for each intervention period and the percent change in CYP before and after the intervention was calculated. The calculation of CYP was based on formulas published previously.^6^ The data collected for condoms and sterilization were not used in calculating CYP because of inconsistencies in recording this information among the four facilities. No statistical analysis was planned.

## 3 RESULTS

The radio discussion panel was held on January 16, 2014, at the Lilongwe Teacher's College in Area 25 and recorded live on Zodiak Broadcasting Station, the largest radio station in Malawi (Table 1). It was attended by more than 100 people, and more than 400 people texted in comments. The panel was comprised of four religious leaders, who promoted family planning use in accordance with their denomination's religious beliefs, quoting passages from the Bible and Quran where helpful. They also answered related questions from the audience and from radio listeners and emphasized the importance of family planning use to maintain healthy mothers, children, and families. Zodiak estimated that approximately 78% of the country's population (10 million Malawians) listened at the time.

The population weekends were held in Kasungu district from January 17 to January 19, 2014, and in Area 25 from January 31 to February 2, 2014, and approximately 45 000 brochures were distributed. The HPP sampled 10 churches and two mosques in Kasungu district and six churches and two mosques in Lilongwe district and found that almost all churches and mosques in the five targeted areas (four areas in Kasungu district and one area in Area 25) participated in the population weekends, with an estimated reach of 350 000 Christians and 5600 Muslims.

The Family Planning Association of Malawi created five family planning community leadership groups in the catchment areas of the intervention sites. These community leadership groups then trained 10 family planning champions, and together with the champions they developed the open days for their catchment area. The Family Planning Association of Malawi estimated that approximately 39 000 people attended their five open day campaigns.

The UNC Project‐Malawi held 16 LARC‐related training sessions at Area 25 Health Center and Kasungu District Hospital between February 2014 and September 2015, training a total of 101 providers. At Area 25 Health Center, two general LARC training courses were held in February 2014, which were then followed by two training courses on immediate PPIUD insertion. Another training course on immediate PPIUD insertion was held in June 2015 for new health providers at Area 25 Health Center. Finally, in September–October 2015, three training courses were held to train 30 providers on Implanon NXT (Merck), which was being introduced into Malawi that fall. Seven providers were selected to mentor the Area 25 trainees. In addition, a Master Family Planning Mentor sponsored by the UNC Project‐Malawi was placed at Area 25 Health Center in April 2014 to help oversee all trainees and family planning activities at the health center. The UNC Project‐Malawi also conducted two training courses in November 2014 in which 30 community health workers from Area 25 Health Center were trained in family planning counseling and SARC provision.^9^

At Kasungu District Hospital, two health providers who attended an immediate‐PPIUD train‐the‐trainers session in February 2014 helped lead two general LARC training sessions in June 2014, which were followed by three 2‐day training courses in both immediate PPIUD insertion and implant insertion. Two additional immediate PPIUD and implant training courses were held in August 2014 and another in November 2014. Seven mentors were selected by the Kasungu district family planning coordinator.

Area 25 Health Center generally had more monthly family planning visits than did Mkanda Health Center (Fig. S1A) in both the pre‐ and post‐intervention periods. However, the number of monthly LARC insertions increased at Area 25 Health Center during the 3‐year monitoring period, but not at Mkanda Health Center (Fig. S1B). Kasungu District Hospital and Dowa District Hospital generally had a similar number of monthly family planning visits in the pre‐intervention period, but there was a notable increase in the number of monthly family planning visits at Kasungu District Hospital during June 2014, when the first round of training courses on interval and immediate postpartum LARC was conducted (Fig. S2A). A similar trend was seen for the number of monthly LARC insertions (Fig. S2B).

**Figure 1:**
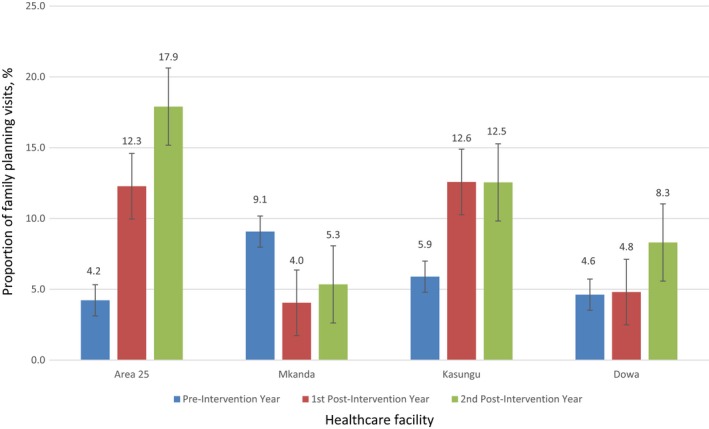
Proportion of family planning visits where a long‐acting reversible contraceptive was inserted at the four health facilities during the three study periods. Error bars represent 95% confidence intervals.

The number of family planning visits at Area 25 Health Center increased by 21.2% from the pre‐intervention year to the second post‐intervention year, whereas it decreased by 6.5% at Mkanda Health Center during the same time period (Table 2). Between the pre‐intervention and second post‐intervention year, the proportion of visits with LARC insertion increased from 4.2% to 17.9%, respectively, at Area 25 Health Center and decreased from 9.1% to 5.3%, respectively, at Mkanda Health Center from the pre‐intervention year to the second post‐intervention year (Fig. 1). At Kasungu District Hospital, the proportion of visits with LARC provision increased from 5.9% in the pre‐intervention year to 12.5% in the second post‐intervention year. Dowa District Hospital had a smaller increase from 4.6% in the pre‐intervention year to 8.3% in the second post‐intervention year.

**Table 2:** Uptake of family planning at Area 25 Health Center and Mkanda Health Center during the pre‐ and post‐intervention years.^a^,^b^

Family planning outcome	Area 25 Health Center				Mkanda Health Center				Difference between the two health centers, %^c^
	Pre‐intervention year	First post‐intervention year	Second post‐intervention year	Difference between pre‐intervention and second post‐intervention years, %	Pre‐intervention year	First post‐intervention year	Second post‐intervention year	Difference between pre‐intervention and second post‐intervention years, %	
Total number of family planning of visits	14 373	17 581	17 430	21.2	5554	6549	5192	–6.5	27.7
Injectable	10 928 (76.0)	14 981 (85.2)	13 323 (76.4)	0.4	4880 (87.9)	6193 (94.6)	4774 (91.9)	4.1	–3.7
Oral contraceptives	1347 (9.4)	435 (2.5)	944 (5.4)	–4.0	104 (1.9)	62 (0.9)	73 (1.4)	–0.5	–3.5
Implant	607 (4.2)	2173 (12.4)	2860 (16.4)	12.2	503 (9.1)	264 (4.0)	277 (5.3)	–3.8	16.0
Postpartum^d^	NA	NA	NA	NA	NA	NA	NA	NA	NA
Intrauterine device	0 (0.0)	101 (0.6)	260 (1.5)	1.5	1 (0.0)	1 (0.0)	1 (0.0)	0.0	1.5
Postpartum^d^	0 (0.0)	11 (0.1)	16 (0.1)	0.1	NA	NA	NA	NA	NA
Sterilization	NA	NA	NA	NA	66 (1.2)	26 (0.4)	67 (1.3)	0.1	NA

Abbreviation: NA, not applicable.

a Values are given as number (percentage) unless indicated otherwise.

b The column percentages were calculated by dividing the number of visits where the contraceptive was given by the total number of visits. The column percentages do not always add up to 100% because condom provision at visits was inconsistently recorded and was therefore excluded from this table.

c Difference between the “Difference between pre‐intervention & second post‐intervention years” columns for Area 25 Health Center and Mkanda Health Center. Positive numbers indicate that Area 25 Health Center had a greater increase for the family planning outcome than did Mkanda Health Center, whereas negative numbers indicate that Mkanda Health Center had a greater increase for the family planning outcome than did Area 25 Health Center.

d Insertion within 48 h of placental delivery.

Immediate postpartum LARC insertion was available at both intervention sites, though uptake was generally low, with only a total of 248 insertions (27 at Area 25 Health Center and 221 at Kasungu District Hospital). 11 (0.1%) women and 16 (0.1%) women at Area 25 Health Center received an immediate postpartum IUD during the first and second post‐intervention years, respectively (Table 2). At Kasungu District Hospital, 201 (1.7%) women and 20 (0.2%) women received an immediate postpartum LARC during the first and second post‐intervention year, respectively (Table 3).

**Table 3:** Uptake of family planning services at Kasungu District Hospital and Dowa District Hospital during the pre‐ and post‐intervention years.^a^,^b^

Family planning outcome	Kasungu District Hospital				Dowa District Hospital				Difference between the two hospitals^c^
	Pre‐intervention year	First post‐intervention year	Second post‐intervention year	Difference between pre‐intervention and second post‐intervention years, %	Pre‐intervention year	First post‐intervention year	Second post‐intervention year	Difference between pre‐intervention and second post‐intervention years, %	
Total number of family planning visits	10 508	11 481	13 169	25.3	7142	8787	9814	37.4	–12.1
Injectable	8027 (76.4)	9482 (82.6)	8933 (67.8)	–8.6	6385 (89.4)	7724 (87.9)	8354 (85.1)	–4.3	–4.3
Oral contraceptives	267 (2.5)	384 (3.3)	2343 (17.8)	15.3	123 (1.7)	166 (1.9)	264 (2.7)	1.0	14.3
Implant	615 (5.9)	1426 (12.4)	1571 (11.9)	6.0	319 (4.5)	419 (4.8)	810 (8.3)	3.8	2.2
Postpartum^d^	0 (0.0)	189 (1.6)	0 (0.0)	0.0	NA	NA	NA	NA	NA
Intrauterine device	4 (0.0)	18 (0.2)	82 (0.6)	0.6	11 (0.2)	3 (0.0)	5 (0.1)	–0.1	0.7
Postpartum^d^	0 (0.0)	12 (0.1)	20 (0.2)	0.2	NA	NA	NA	NA	NA
Sterilization	20 (0.2)	137 (1.2)	135 (1.0)	0.8	298 (4.2)	437 (5.0)	179 (1.8)	–2.4	3.2

Abbreviation: NA, not applicable.

a Values are given as number (percentage) unless indicated otherwise.

b The column percentages were calculated by dividing the number of visits where the contraceptive was given by the total number of visits. The column percentages do not always add up to 100% because condom provision at visits was inconsistently recorded and was therefore excluded from this table.

c Difference between the “Difference between pre‐intervention & second post‐intervention years” columns for Kasungu District Hospital and Dowa District Hospital. Positive numbers indicate that Kasungu District Hospital had a greater increase for the family planning outcome than did Mkanda Health Center, whereas negative numbers indicate that Dowa District Hospital had a greater increase for the family planning outcome than did Area 25 Health Center.

d Insertion within 48 h of placental delivery.

At all health facilities except Mkanda Health Center, CYP increased between the pre‐intervention year and the second post‐intervention year (Table 4). The increase in CYP was greatest at Area 25 Health Center, with an overall increase of 175.1%, compared with a 33.8% decline at Mkanda Health Center. Kasungu District Hospital had an overall increase in CYP of 90.7%, compared with an increase of 64.4% at Dowa District Hospital.

**Table 4:** Couple years of protection at the four health facilities

Health facility	Pre‐intervention year	First post‐intervention year	Second post‐intervention year	Change overall, %^a^
Area 25 Health Center	5061	10 734	13 925	175.1
Mkanda Health Center	3143	2526	2082	–33.8
Kasungu District Hospital	4303	7740	8204	90.7
Dowa District Hospital	2787	3323	4581	64.4

a Change from the pre‐intervention year to the second post‐intervention year.

## 4 DISCUSSION

The present family planning interventions contributed to a greater increase in CYP at the two targeted sites when compared with their comparison sites. Much of the increase in CYP at the two intervention sites was attributable to an increased uptake of LARC, particularly implants, indicating that the contraceptive method mix at those sites was also improved. It is important to note that the demand generation activities did not focus on LARC use but on the importance of family planning use in general. Therefore, the increase in LARC uptake is likely attributable to the increased access to LARC offered by the providers at the intervention sites after the LARC training courses because many providers were not counseling about or offering LARC prior to the training because of their lack of skills to insert and remove these contraceptives.

The present interventions included community mobilization, which helped to increase the demand for family planning use. Uptake of family planning is often low because of misinformation regarding the adverse effects of contraceptives.^3^, ^10^, ^11^ The open days provided correct information about family planning and involved the education of traditional leaders, who are custodians of culture and influence decision‐making at both the community and the family level. Other programs in Africa have also successfully involved traditional leaders in health campaigns, although none focused on family planning only.^12^, ^13^, ^14^

In addition, local religious leaders were educated about the benefits of family planning. The religious leaders were generally supportive of undertaking the events in their communities, including learning about the benefits of family planning and disseminating this information to their congregations. A similar study in Jordan also worked with religious leaders and found that trained religious leaders were more effective in disseminating family planning messages compared with untrained counterparts.^15^ Other interventions in Africa that have focused on involving religious leaders in family planning messaging have also found positive results.^16^, ^17^, ^18^, ^19^

Skilled healthcare providers are critical in family planning programs to meet the supply‐side needs for family planning provision. Inadequately trained staff could be a barrier to the provision of family planning, especially LARC.^20^, ^21^ Therefore, the present intervention included staff training and on‐the‐job mentoring after the courses to ensure the staff became competent. This strategy was also successfully adopted in Kenya and Senegal, where investigators have recommended that staff training and mentoring be included in family planning programs to increase the uptake of family planning, especially LARC.^22^, ^23^

Postpartum women are among those with a high unmet need for family planning.^24^ Studies have shown that postpartum women want to avoid pregnancy in the next 24 months, but 70% of them are not using contraceptives.^24^, ^25^ Improving postpartum access to family planning, especially LARC, could therefore help to reduce the unmet need for family planning. In the present study, PPIUD was offered at Area 25 Health Center, whereas both PPIUD and immediate postpartum implants were offered at Kasungu District Hospital. However, uptake of these services, particularly PPIUD use, was generally low. There were multiple challenges in implementing PPIUD services because only few women were interested in this method, and it was difficult to time their deliveries with the training courses and times when the family planning mentors were available. Frequent staff transfers between health facilities also made it difficult to have all staff trained at all times at the targeted facilities.

Because the family planning data were collected from the government family planning registers and DHIS2, it was only possible to collect information that was recorded in them. There were some inconsistences in filling the registers at the health facilities. For example, data on the age and parity of the family planning patients were inconsistently collected, so this information could not be analyzed. Also, finding a pure comparison facility for the intervention sites was a challenge because other organizations may have been implementing interventions in these sites that could have affected their family planning provision. However, we used the most similar comparison facilities that we could find. An additional limitation is that the radio discussion panel was broadcast nationally, and therefore the populations in the comparison communities were also exposed to this component. Given these limitations, it was not possible to examine causal relationships, and therefore the findings from the present analysis demonstrate changes before and after program intervention activities.

In conclusion, after implementation of a package of family planning interventions, the uptake of family planning, particularly LARC, increased at the intervention facilities, with a corresponding increase in CYP. To increase family planning uptake, programs should strive to provide correct information on contraceptives through community mobilization and ensure the availability and skilled provision of all methods so that patients can use the contraceptive of their choice. Future family planning programs should focus on scaling up these demand‐creation and supply‐improvement interventions at other health facilities and improving prenatal family planning counseling to increase the uptake of immediate postpartum LARC.

## AUTHOR CONTRIBUTIONS

CL contributed to the data analysis and writing the manuscript. NK, BP, OM, NC, and JHT contributed to implementing the interventions, data collection, and revising the manuscript. ISS and KS contributed to data analysis and interpretation, and revising the manuscript. All authors read and approved the final manuscript.

## CONFLICTS OF INTEREST

The authors have no conflicts of interest.

## Supporting information


**Figure S1.** Number of family planning visits (a) and long‐acting reversible contraceptive (LARC) insertions (b) per month at Area 25 Health Center and Mkanda Health Center during the pre‐ and post‐intervention periods.

**Figure S2.** Number of family planning visits (a) and long‐acting reversible contraceptive (LARC) insertions (b) per month at Kasungu District Hospital and Dowa District Hospital during pre‐ and post‐intervention periods.
