# Supplementary material for: Effect of family planning interventions on couple years of protection in Malawi
Source: Int J Gynaecol Obstet. 2018 Feb 2;141(1):37–44. doi: 10.1002/ijgo.12439 (PMC5873398; doi:10.1002/ijgo.12439)
Supplement: Supplementary file 1 — Figure S1. Number of family planning visits (a) and long‐acting reversible contraceptive (LARC) insertions (b) per month at Area 25 Health Center and Mkanda Health Center during the pre‐ and post‐intervention periods. [file IJGO-141-37-s001.docx]

F

D

C

B (full Interventions begin)

E
