# Supplementary material for: Effect of family planning interventions on couple years of protection in Malawi
Source: Int J Gynaecol Obstet. 2018 Feb 2;141(1):37–44. doi: 10.1002/ijgo.12439 (PMC5873398; doi:10.1002/ijgo.12439)
Supplement: Supplementary file 3 — Figure S2. Number of family planning visits (a) and long‐acting reversible contraceptive (LARC) insertions (b) per month at Kasungu District Hospital and Dowa District Hospital during pre‐ and post‐intervention periods. [file IJGO-141-37-s003.docx]

D

A

C

B (full Interventions begin) (B)
