# Supplementary material for: Effect of family planning interventions on couple years of protection in Malawi
Source: Int J Gynaecol Obstet. 2018 Feb 2;141(1):37–44. doi: 10.1002/ijgo.12439 (PMC5873398; doi:10.1002/ijgo.12439)
Supplement: Supplementary file 4 [file IJGO-141-37-s004.docx]

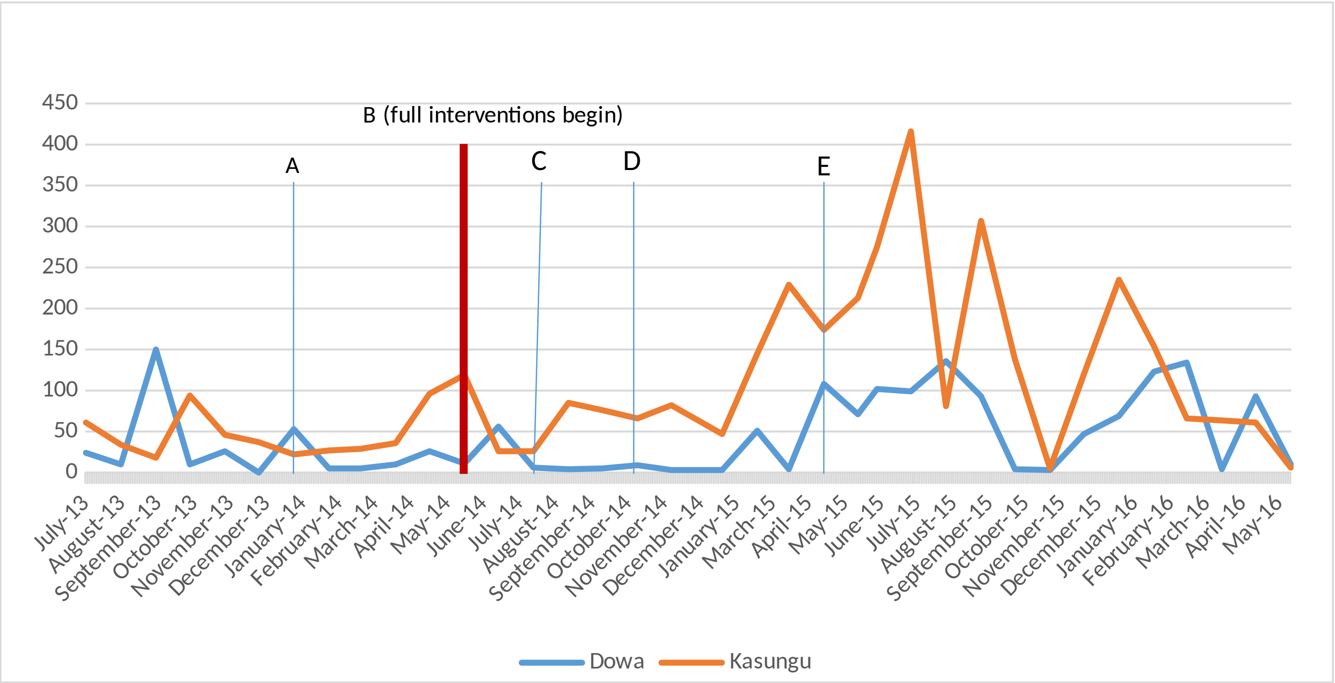

B (full interventions begin)
A
C
D
E
450
400
350
300
250
200
150
100
50
0
July-13
August-13
September-13
October-13
November-13
December-13
January-14
February-14
March-14
April-14
May-14
June-14
July-14
August-14
September-14
October-14
November-14
December-14
January-15
March-15
April-15
May-15
June-15
July-15
August-15
September-15
October-15
November-15
December-15
January-16
February-16
March-16
April-16
May-16
Dowa
Kasungu
